# Antioxidative Characteristics and Sensory Acceptability of Bread Substituted with Purple Yam (*Dioscorea alata* L.)

**DOI:** 10.1155/2021/5586316

**Published:** 2021-07-14

**Authors:** Siti Tamaroh, Ajat Sudrajat

**Affiliations:** Department of Agroindustry, Universitas Mercu Buana Yogyakarta, Yogyakarta 55753, Indonesia

## Abstract

In this study, plain bread was made by substituting wheat flour with purple yam flour (*Dioscorea alata*, L). The addition of 0, 10, 15, 20, and 30% purple yam flour aims to increase the functional value of plain bread as a source of natural antioxidants. The bread produced with two baking temperatures (170°C and 180°C) was tested for anthocyanin levels, total phenol, antioxidant activity (DPPH free radical scavenging), volume expansion, color, and preference test. The results showed that the temperatures of the bread baking did not affect anthocyanin levels, total phenol, antioxidant activity, volume expansion, and bread color (*p* > 0.05). The substitution of purple yam flour had a significant effect on anthocyanin levels, total phenol, antioxidant activity, volume, and bread color (*p* < 0.05). The levels of anthocyanins, total phenol, antioxidant activity, and darker color increased with the addition of purple yam flower whereas the rate of expansion reduced. However, the addition of purple yam flour did not affect the level of preference for the bread produced. Purple yam flour can be added to the manufacture of bread made from wheat flour as much as 30% with a baking temperature of 180°C. The resulting bread contained total anthocyanins 54.62 mg/100 g db, total phenol 391.14 mg EAG/100 g db, antioxidant activity 48.53% and volume expansion 64.85%, color *L*^∗^ = 53.06, *a*^∗^ = 6.52, *b*^∗^ = 13.32, *C* = 14.87, *H* = 64.06, and sensory score = 3.24 (preferred).

## 1. Introduction

The bakery industry in Indonesia is developing rapidly. This is triggered by an increasing middle-class population, high income of young people, and changing consumption patterns of people who adopt an urban lifestyle. The consumption of plain bread has increased gradually (0.062 packs/cap/year in 2014 to 0.37 packs/cap/year in 2017) [1]. The consumption of bread in the world has also increased because bread is a nutritious and practical food [2]. Wheat flour is the main ingredient used in plain bread. National wheat flour consumption in 2019 is estimated to reach 8 million metric tons (mt). This number will continue to grow as Indonesia's population grows. Meanwhile, wheat flour has become an important food ingredient for Indonesians to produce a wide variety of food products. The demand for wheat imports in Indonesia increased by around 9% to 11.48 million tons in 2017 [3]. This is due to the increasing demand for the domestic food industries to cover the consumption needs of wheat-based products in Indonesia. In 2019, the need for wheat imports is estimated to grow 5% from the imports in 2018 of 10.09 million tons [3].

Diversification of agricultural-based products is important to increase the use of local sources of an agriculture product. This will divert consumption of imported wheat-based food product into local-based food product and reduce demand for wheat flour. In the end, this will impact on achieving food security and fulfilling nutrition based on food resources and local wisdom by developing local food in various regions in Indonesia. Several studies have conducted on food diversification efforts to reduce the use of wheat flour. For example, made bread substituted with flour Chinese yam (*Dioscorea opposita* Thunb.) recommended that substitution can be made up to 15% [4]. Substituted bread with sweet potato flour (*Ipomoea batatas* L.) up to 15% reported no reduction in the nutritional quality and acceptance [5]. Research conducted by [6], who made bread substituted with flour finger millet (*Eleusine coracana*) up to 20%, found acceptable sensory qualities and physical properties.

Purple yam tubers are one of the local potential natural sources that have not been explored. Purple yam (*Dioscorea alata* L.) is a plant that can withstand the conditions where it grows with minimal nutrients. The nutritional components contained in purple yam are carbohydrates (17.10-29.37%), protein (1.29-3.00%), fat (0.29%), and fiber (6.70-11.62%) and ash (0.85-1.44%) with a moisture content of 65.47-82.46% [7]. The mineral components contained in purple yam are relatively complete, such as K (224.54-483.21 mg/100 g), Ca (15.63-61.97 mg/100 g), Mg (16.75-43.06 mg/100 g), Fe (1.40-13.40 mg/100 g), Zn (0.43-2.83 mg/100 g), and P (329.37-699.91 mg/100 g). Purple yam has the potential as a source of natural anthocyanins, which can be used as antioxidants and natural food coloring. The level of purple yam anthocyanin is 31 mg/100 g of dry matter [8]. The use of yam is still limited to traditional food processing in Indonesia (steamed, fried, baked, and made into chips) [9]. Yam can be processed into flour with several advantages, such as easy to store, has a small volume, easy to transport, and more flexible for various processed food products.

Research on the manufacture of purple sweet potato flour has been carried out, with high anthocyanin content (82.72 mg/100 g) [10]. The purple yam flour has the potential to be a substitute for fresh bread making which has the potential to be a source of antioxidants and reduce the need for imported wheat flour. In this study, we made plain bread substituted with purple yam flour, so that it is expected to produce bread with the benefits of having natural antioxidants which are preferred and are expected to suppress the use of wheat flour as the main ingredient in making plain bread.

## 2. Materials and Methods

### 2.1. Material

The purple yam tubers (*Dioscorea alata* L.) and wheat flour with high protein content were obtained from Bantul, Yogyakarta (Cakra Kembar, Bogasari, Indonesia). The chemicals diethyl ether (technical), DPPH (2,2-diphenyl-1-picrylhidrazil), ethanol (technical), methanol (technical), KCl, sodium nitrate, buffer Na-acetic acid, sodium hydroxide. (Merck KGaA, 64271 Darmstadt, Germany), sodium acetate, HCl, gallic acid (Sigma Chemical Co., St Louis, United States), and Folin-Ciocalteu reagent were purchased from Sigma.

### 2.2. Purple Yam Flour Preparation

The purple yam flour was prepared according to the method developed by Tamaroh et al. [10]. The purple yam flour was peeled, sliced, and blended for 8 min and dried in a cabinet drier (local fabricated) at 50°C for 10 h until a moisture content of 10% was achieved. The dried samples were grounded into powder and sieved with an 80-mesh sieve. The purple yam flour obtained was ready to be used for further analysis.

### 2.3. Plain Bread Preparation

Bread making was carried out according to the method developed by Devani et al. [6].  Table 1 shows the formulation. The addition of purple yam flour was conducted at percentage of 0%, 10%, 15%, 20%, and 30%.

Yeast was added to cold water (temperature 20°C), left to stand for 10 min, and referred to as dough A. Wheat flour, eggs, sugar, full cream milk, bread improver, and yam flour were mixed at various ratios and referred to as dough B. Dough A and dough B were mixed and added with margarine and salt. The dough mixture was carried out by the mixing process in order to obtain a smooth-textured dough and let stand for 40 min. Next, the dough was put into the mould, before baking, let stand for 60 min. Baking was carried out for 25 min with temperature variations of 170°C and 180°C. The bread produced was tested for moisture content, anthocyanin content, total phenolic content, antioxidant activity, color, volume expansion, and sensory evaluation.

### 2.4. Determination of Total Anthocyanins

Total anthocyanins were determined by the method proposed by Giusti and Wrolstad [11]. A total of 0.4 mL of extract was put in 2 test tubes. The first test tube was added with a buffer of potassium chloride (0.025 M) pH 1 of 2.6 mL. The second test tube was added with sodium acetate buffer solution (0.4 M) at 4.5 of 2.6 mL. The absorbance of the two samples was measured with a spectrophotometer at wavelengths of 520 and 700 nm after standing for 15 min. The absorbance value was calculated by the formula *A* = (A520 − A700) pH 1 − (A520 − A700) pH 4.5. The anthocyanin concentration was calculated as cyanidin-3-glycoside using a molar extinction coefficient of 26,900 L cm^−1^ and a molecular weight of 484.82. Anthocyanin concentration (mg/L) = (A × BM × FP × 1000)/(*e* × 1), where *A* is the absorbance, BM is the molecular weight (484.82), FP is the dilution factor (3 mL/0.4 mL), and *e* is the molar extinction coefficient (26,900 L cm^−1^).

### 2.5. Determination of Total Phenolic Content

Total phenolic levels were determined by the Folin-Ciocalteu method proposed by Roy et al. [12] using gallic acid as the standard. A total of 50 *μ*L of sample was added with 250 *μ*L of Folin-Ciocalteu solution, then let stand for 1 min and added 750 *μ*L of 20% NaCO_3_, then pulverized, and added aquades to a volume of 5 mL. After incubation for 5 min at room temperature, the absorbance was measured at *λ* 760 nm (Spectrophotometer UV mini 1240 UV-Vis merk Shimadzu, German). Gallic acid was used as a standard, and a calibration curve was made with gallic acid 31.875 to 510 mg/L with *r* = 0.99. The calculation result of total phenolic content was expressed as mg gallic acid equivalent (EAG) per gram of dry extract.

### 2.6. DPPH Method of Free Radical Scavenging Test

The antioxidant activity test was carried out by knowing the capacity of DPPH free radical scavenging. Around 0.2 mL of the sample plus 3.8 mL of 0.1 mM DPPH solution was vortex (Vortex Mixer, VM-300, Iowa USA) for 3 min and observed the absorbance at 30 minutes with a spectrophotometer (UV mini 1240 UV-Vis merk Shimadzu, German) at a wavelength of 517 [13]. The calculation of the scavenging capacity of free radicals was calculated and expressed in percent (%) RSA (% radical scavenging activity) which was the % of DPPH bleaching.

(1) $$\begin{array}{c} \%\text{RSA}=1-\frac{\left(\text{absorbance of sample}\right)}{\left(\text{absorbance of control}\right)}\times 100\%. \end{array}$$

### 2.7. Bread Volume Expansion Test

Measurement of bread expansion was carried out using the rape seed displacement method proposed by Matz [14]. In this study, the seeds used were Setaria italica seeds. The volume of bread expansion was measured by comparing the increase in the volume of bread after baking with the volume of bread before baking. Volume measurement was done by inserting grain in a dough mold until the surface was flat, after which the grain was measured in volume with a measuring cup. Furthermore, the volume of the dough before being baked using a known volume mold was measured; then, the dough was put in the mold and filled with grains to the full extent and the volume was recorded. Furthermore, the measurement of the volume of bread that has been oven carried out by inserting the grains in the container containing the bread until the full limit mark; then, the grains were measured in the measuring cup. The volume of bread expansion is measured by the ratio between the volume of bread before baking and the volume of bread after baking as the percent expansion.

### 2.8. Sensory Test

The sensory test was carried out using the Hedonic scale method proposed by Mitiku et al. [5]. The assessment was carried out by 25 panelists with an interval scale from 1 to 5 (1: very disliked, 2: disliked, 3: liked, 4: preferred, and 5: very liked). The attributes assessed were color, aroma, texture, taste, and overall acceptance.

### 2.9. Statistical Analysis

The experimental design used was a two-way design with the addition of purple yam flour (0, 10, 15, 20, and 30%) and baking temperatures (170°C and 180°C). All data analyses were carried out in duplicate. Data were expressed as mean ± SD. The data obtained were tested statistically by the SPSS method and if significantly different, then continued with the “Duncan new multiple range test” (DMRT) at the 5% degree of confidence.

## 3. Results and Discussion

### 3.1. Anthocyanin Levels

**Figure 1:**
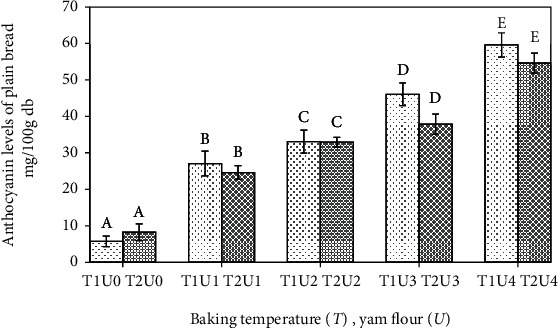
Anthocyanin levels of plain bread with different baking temperature and yam flour addition. Note: T1: baking temperature 170°C; T2: baking temperature 180°C; U0: yam flour 0%; U1: yam flour 10%; U2: yam flour 15%; U3: yam flour 20%; U4: yam flour 30%. The letter above the bar graph beside the same number shows that it is not different based on the DNMRT statistical test at the level of confidence 5%.

The statistical test results of anthocyanin levels showed that the baking temperature had no effect on anthocyanin levels (*p* > 0.05), while the treatment of adding the purple yam flour had an effect on the anthocyanin levels of the fresh bread (*p* < 0.05). A study of Tamaroh et al. [10] showed that the anthocyanin level in the purple yam flour is 82.72 mg/100 g db. Another latest study by Ratnaningsih et al. [15] showed that the anthocyanin content in purple yam flour is 74.3 mg/100 g. The anthocyanin value of the eight plain bread formulated with purple yam flour increased (*p* < 0.05). The anthocyanin levels of plain bread in this study can be seen in Figure 1.

The highest anthocyanin level (59.58 mg/100 g db) was found in plain bread with the addition of 30% purple yam flour at baking temperature of 170°C which was not statistically different from the anthocyanin levels of 54.62 mg/100 g at baking temperature of 180°C. Another latest study by Bartl et al. [16] showed that the anthocyanin levels in the bread formulated using blue and purple wheat varieties ranged from around 53.1 mg/100 g (db) to 52.00 mg/100 g (db), respectively. The results obtained in this study were not much different from their study. The anthocyanin levels of plain bread increased with the addition of wheat flour with purple yam flour. The anthocyanin content of the bread did not decrease with the increment of baking temperature. In a study by Srivichai and Hongsprabhas [17], they reported that the majority of anthocyanins found in purple yam flour are acylated anthocyanins (alatanin C (cyanidin 3-(6-sinapoyl gentiobioside) and cyanidin or peonidin monoacylated and diacylated with sinapic or ferulic acid). A study by Bakowska-Barczak et al. [18] stated that the type of acylated anthocyanins is heat resistant. In a study by Lachman et al. [19], they are reported in purple potatoes where baking increased anthocyanins up to 3.34 times. In a study by Murador et al. [20], the baking process increased anthocyanin levels in vegetables.

### 3.2. Total Phenol

**Figure 2:**
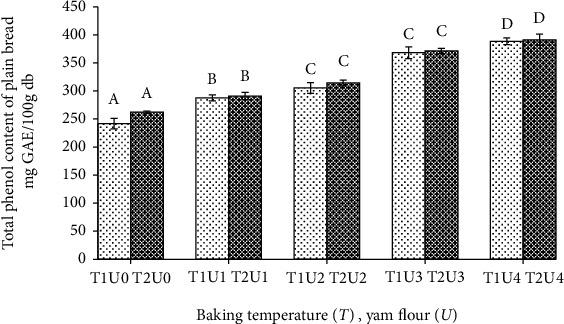
Total phenol content of plain bread with different baking temperature and yam flour addition. Note: T1: baking temperature 170°C; T2: baking temperature 180°C; U0: yam flour 0%; U1: yam flour 10%; U2: yam flour 15%; U3: yam flour 20%; U4: yam flour 30%. The letter above the bar graph beside the same number shows that it is not different based on the DNMRT statistical test at the level of confidence 5%.

The results of the phenol content in the formulated plain bread showed that the baking temperature treatment had no effect on the phenol content (*p* > 0.05), while the addition of purple yam flour had an effect on the phenol content (*p* < 0.05). Results for the total phenol content of plain bread can be seen in Figure 2.

Figure 2 shows that the highest total phenol content (391.14 mg GAE/100 g db) was found in plain bread with baking at 180°C and 30% of purple yam flour. This result is not significantly different from the baking temperature of 170°C (388.43 mg GAE/100 g db). The total phenol content of plain bread further increased with the addition of yam flour. Studies of [10] showed the total phenol content in purple yam flour (454.67 mg GAE/100 g db). In a previous study, Hong and Koh [21] stated that the baking processing method has no effect on the total phenolic content of purple sweet potato. In another study by Liu et al. [22], they observed bread making substituted with purple sweet potato flour and stated that the addition of purple sweet potato flour 15% significantly increased total phenol compounds and their antioxidant activity. They reported total phenol of 224 mg GAE/100 g db. In a study by Ming et al. [4], they have reported that the substitution up to 15% of a tuber *Dioscorea opposita* Thunb. flour produces quality bread and is a source of antioxidants and total phenol content is 210.93 mg 211 GAE/100 g db.

### 3.3. Antioxidant Activity

**Figure 3:**
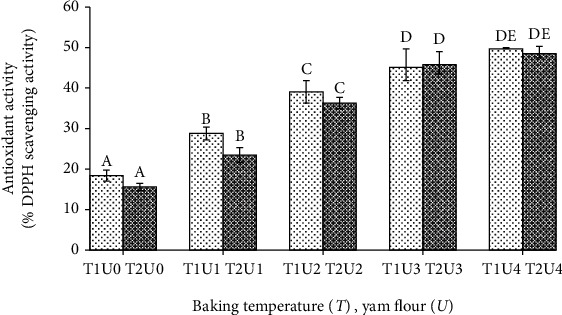
Antioxidant activity of plain bread with different baking temperature and yam flour addition. Note: T1: baking temperature 170°C; T2: baking temperature 180°C; U0: yam flour 0%; U1: yam flour 10%; U2: yam flour 15%; U3: yam flour 20%; U4: yam flour 30%. The letter above the bar graph beside the same number shows that it is not different based on the DNMRT statistical test at the level of confidence 5%.

The results for antioxidant activity of formulated bread showed that the baking temperature treatment had no effect on the antioxidant activity (*p* > 0.05), while the addition of purple yam flour had a significant effect (*p* < 0.05). The results for the antioxidant activity of plain bread can be seen in Figure 3.

The antioxidant activity found in this study was shown as the percent inhibition of DPPH free radicals. Figure 3 shows that the greater the addition of purple yam flour, the greater the antioxidant activity. In the treatment of adding 20% purple yam flour to bread making, it was not significantly different from the addition of 30% purple yam flour. In this study, the components of anthocyanins and phenolic compounds that contribute to antioxidants are the components of antioxidants.

The antioxidant activity of DPPH free radical scavenging in purple yam flour was 83.68% [23]. In a study by Larief et al. [24] on sponge bread from wheat flour and white rice flour substituted with 30% purple yam flour showed antioxidant activity of DPPH free radical binding of 26.9%. Another latest study by Curayag et al. [25] showed that the substitution of purple sweet potato flour increased antioxidant activity. They reported that the antioxidant activity of DPPH free radical binding of 10.14% with 20% substitution. In this study, the antioxidant activity of bread with purple yam flour substitution ranged from 23.43 to 49.70%.

Studies of [26] showed a positive correlation between total phenols and anthocyanins on antioxidant activity in mushroom. Total phenol compounds and anthocyanins are closely related to antioxidant activity [27, 28]. Teow et al. [29] stated that total phenol compounds were related to the antioxidant activity of purple sweet potato flour. In a study by Jing et al. [30], they reported that phenolic compounds have a positive correlation with scavenging activity and the reducing power of DPPH compounds. In a study by Tamaroh et al. [31], total anthocyanins and phenol compounds in purple yams treated with blanching have a positive correlation on the antioxidant activity of DPPH free radical scavenging. Lachman et al. [19] stated that total anthocyanins had a positive correlation with antioxidant activity.

### 3.4. Volume Expansion of Bread

The expansion of the volume of plain bread is the ratio between the volume of bread before and after baking. The volume of bread expansion is caused by the ability of the dough to form and hold the gas produced during fermentation. In the baking process, gluten is coagulated so that it becomes stiffer and the volume of the bread does not shrink anymore because of the hollow structure that forms inside the bread.

**Figure 4:**
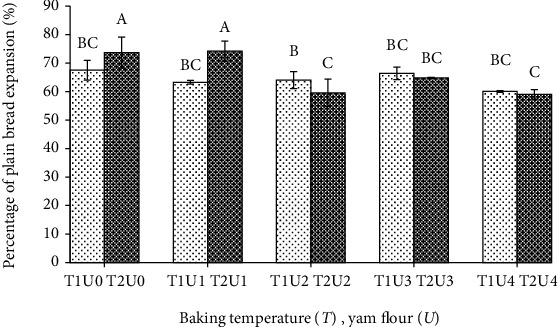
Percentage of plain bread expansion with different baking temperature and yam flour addition. Note: T1: baking temperature 170°C; T2: baking temperature 180°C; U0: yam flour 0%; U1: yam flour 10%; U2: yam flour 15%; U3: yam flour 20%; U4: yam flour 30%. The letter above the bar graph beside the same number shows that it is not different based on the DNMRT statistical test at the level of confidence 5%.

The results of statistical tests on the expansion volume of plain bread showed that the addition of purple yam flour had a significant effect on expansion (*p* < 0.05), but the baking temperature had no effect (*p* > 0.05). Bread without purple yam flour and the addition of 10% purple yam flour which was baked at 180°C shows the expansion of bread greater than baking at 170°C. This can be explained that at a baking temperature of 180°C, there has been completed gelatinization of the gluten dough, so that the gluten network can hold the gas formed during baking. The temperature of baking bread is in accordance with the finding reported by Miñarro et al. [32], who made bread without gluten and baking was carried out at 180°C for 25 min. The results for the expansion volume of plain bread can be seen in Figure 4.

**Figure 5:**
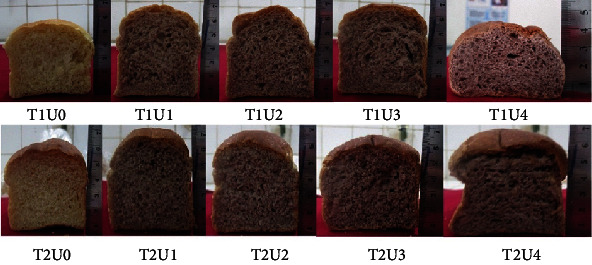
Appearance of bread with different baking temperature and yam flour addition. Note: T1: baking temperature 170°C; T2: baking temperature 180°C; U0: yam flour 0%; U1: yam flour 10%; U2: yam flour 15%; U3: yam flour 20%; U4: yam flour 30%.

Figure 4 shows the expansion volume of bread substituted with yam flour 30%, at baking temperature of 170°C and 180°C, did not differ from the bread without yam flour which was baked at 170°C. The volume expansion of bread is influenced by the gluten component in wheat flour. In the baking process, gluten is coagulated so that it becomes stiffer and the volume of the bread does not shrink anymore due to the hollow structure that forms inside the bread [4]. Plain bread with high gluten content causes the bread to become elastic and is able to form bread structures by holding gas. The decrease in the volume of the bread produced is influenced by the decrease in the gluten content of the flour so that the volume of the dough that previously expanded during fermentation tends to decrease when baked. Bread made with the addition of 30% purple yam flour, the volume was found to be low because of the low gluten component in the formula. The low gluten component results in the formation of low gluten networks so that it will reduce the stability of the dough to trap CO_2_ gas that is formed during baking [33]. The results obtained are in line with the results reported by Pycia and Ivanišová [34], who studied bread substituted with nuts, produced bread with little expansion, due to the lack of gluten proportion in the bread formula that is replaced by bean flour. A similar trend was observed in the addition of other flours such as with tuber flour *Dioscorea* opposita Thunb. by Li et al. [4] which reported the gas holding capacity of bread had decreased during proofing and baking. This decrease would be due to the destruction of gluten by adding yam flour. The volume expansion of substituted bread will decrease due to a decrease in the capacity to capture gas that is formed during baking as the effect of the gluten destruction [2]. The appearance of the bread is shown in Figure 5.

Figure 5 supports the results of statistical tests that the addition of purple yam flour affects the expansion volume of plain bread. The expansion volume of plain bread shows that the expansion volume of bread containing 30% of purple yam flour, at the baking temperature of 170°C and 180°C, did not differ from the control treatment which was baked at 170°C. The expansion of plain bread with 30% of purple yam flour, baked at 180°C, differs from the control treatment which was baked at 180°C. Plain bread with 30% of purple yam flour, baked at 180°C, show an imperfect level of expansion volume.

### 3.5. Bread Color

The results for the color of the plain bread are shown in Table 2. The value for the letter *L*^∗^ indicates changes in brightness or lightness [35]. The addition of purple yam flour caused low *L*^∗^ value, which indicated that the plain bread was getting darker in color. A similar trend in color was found by Azni et al. [36], who made cookies substituted with purple sweet potato paste. Their results showed that the *L*^∗^ color value decreased, which indicates that the product was getting darker. In a study by Souripet [37], they reported the *L*^∗^ color value of purple sweet potato rice decrease when more purple sweet potato was added. In another study by Selvakumaran et al. [38], they reported that substitution of wheat flour with orange sweet potato (*Ipomoea batatas*) puree in brownie formulations significantly decreased lightness (*L*^∗^).

The *a*^∗^ value indicates the chromatic color for red. In this study, the *a*^∗^ value did not show a significant difference (*p* > 0.05). The value of *b*^∗^ represents the chromatic blue color, decreased with the addition of purple yam flour indicative of the increasing blueness of plain bread. The increasing blueness of plain bread shows an increase anthocyanins and total phenol. The results of the present study corroborate with the findings of Liu et al. [2] which reported the *b*^∗^ values of breads decreased with increasing amounts of purple yam flour in bread. In a study by Azni et al. [36], they reported the change in *b*^∗^ color indicates the presence of anthocyanin and phenol components.

The *C* value or the chroma values indicates the color intensity of samples perceived by humans. The higher the chroma values, the higher the color intensity of samples [39]. The *C* value in this study showed a decrease with the addition of purple yam flour in the plain bread, which means that the intensity of the bread produced decreased in color. This can be explained that the bread substituted with purple yam flour shows the presence of anthocyanins and phenol components which cause a decrease in color intensity.

Hue angle (*H*), considered the qualitative attribute of color, is the attribute according to which colors have been traditionally defined as reddish or greenish [39]. In this study, the *H* value shows a decrease with the addition of purple yam flour in the plain bread. This shows that the addition of purple yam flour will cause the bread to be more purple which indicates it is the presence of anthocyanin and phenol components.

### 3.6. Sensory Evaluation of Bread

The results obtained in this study were carried out by the sensory evaluation test using the Hedonic 5-scale method. The intervals for the assessment of the liking test were as follows: 1: very disliked, 2: disliked, 3 liked, 4: preferred, and 5: very liked. The results of the preference test can be seen in Table 3.

Table 3 shows the acceptance values for color, aroma, texture, taste, and overall acceptance of plain bread. The acceptance of the color, aroma, and texture showed no significant difference (*p* > 0.05). There was a significant difference observed in texture acceptance which indicates that the addition of purple yam flour reduced the acceptance at 170°C. However, at baking temperature of 180°C, the acceptance was not significantly different. Bread substituted with purple yam flour and baking temperature did not give any significant difference in taste acceptance (*p* > 0.05). The overall acceptance value showed that the addition of purple yam flour and baking temperature was not significantly different (*p* > 0.05). The average value of acceptance of the bread produced was 3.28 which indicates the panelists like the bread. The overall acceptance of plain bread supports acceptable values for color, aroma, texture, and taste.

## 4. Conclusion

The results of this study indicate that plain bread made with wheat flour substituted with purple yam flour increased levels of anthocyanins, total phenol, and antioxidant activity whereas decreased the volume expansion rate. Baking temperature generally does not affect several chemical and physical parameters of bread. At the baking temperature of 180°C, there has been completed gelatinization of the gluten dough, so that the gluten network can hold the gas formed during baking. Bread with the addition of 30% purple yam flour, roasting at 180°C, resulted in good bread volume development and high antioxidant activity. The characteristics of bread with 30% yam flour substitution and 180°C baking temperature were found to have total anthocyanin 54.62 mg/100 g, total phenol 391.14 mg EAG/100 g, antioxidant activity 48.53% (free radical inhibition DPPH) and the percentage of volume expansion 64.85%, color *L*^∗^ = 53.06, *a*∗ = 6.52, *b*^∗^ = 13.32, *C* = 14.87, *H* = 64.06, and Hedonic score = 3.24 (preferred). Based on the results obtained in this study, substitution of yam flour can be done up to 30%.

## Figures and Tables

**Table 1:** Ingredient formulation for plain bread with different yam flour addition.

Ingredients	Bread U0	Bread U1	Bread U2	Bread U3	Bread U4
	(0%)	(10%)	(15%)	(20%)	(30%)
Wheat flour (%)	47.08	42.37	40.02	37.66	32.96
Purple yam flour (%)	0.00	4.71	7.06	9.42	14.12
Egg (%)	4.90	4.90	4.90	4.90	4.90
Table sugar (%)	4.71	4.71	4.71	4.71	4.71
Full cream milk (%)	2.35	2.35	2.35	2.35	2.35
Bread improver (%)	0.38	0.38	0.38	0.38	0.38
Salt (%)	2.35	2.35	2.35	2.35	2.35
Margarine (%)	11.30	11.30	11.30	11.30	11.30
Yeast (%)	1.51	1.51	1.51	1.51	1.51
Cold water (%)	25.42	25.42	25.42	25.42	25.42

**Table 2:** Color of plain bread with different baking temperature and yam flour addition.

Color	Baking temperature (°C)	Bread U0 (0%)	Bread U1 (10%)	Bread U2 (15%)	Bread U3 (20%)	Bread U4 (30%)
*L* ^∗^	T1 (170)	66.83 ± 4.67^b^	59.40 ± 6.92^ab^	55.68 ± 6.31^a^	57.42 ± 6.12^a^	61.63 ± 4.24^a^
	T2 (180)	66.45 ± 4.15^b^	58.53 ± 2.45^ab^	57.40 ± 4.46^a^	52.96 ± 6.37^a^	53.06 ± 5.22^a^
*a* ^∗^	T1 (170)	5.96 ± 0.14	5.90 ± 0.57	5, 96 ± 1, 16	5.55 ± 0.47	5.75 ± 0.13
	T2 (180)	4.99 ± 1.32	6.05 ± 0.71	6, 04 ± 0, 84	6.75 ± 1.79	6.52 ± 1.40
*b* ^∗^	T1 (170)	18.78 ± 1.23^a^	15.45 ± 0.75^b^	14.71 ± 0.89^b^	14.84 ± 0.73^b^	14.92 ± 1.46^b^
	T2 (180)	18.84 ± 4.56^a^	14.62 ± 1.39^b^	14.16 ± 1.15^b^	13.53 ± 0.05^b^	13.32 ± 0.17^b^
*C*	T1 (170)	19.68 ± 1.20^a^	15.71 ± 0.27^b^	15.86 ± 1.30^b^	15.69 ± 0.62^b^	15.99 ± 1.40^b^
	T2 (180)	19.42 ± 3.84^a^	15.84 ± 1.55^b^	15.06 ± 0.92^b^	15.16 ± 0.84^b^	14.87 ± 0.47^b^
*H*	T1 (170)	72.36 ± 0.68^a^	69.12 ± 0.92^b^	68.01 ± 2.66^b^	69.54 ± 0.66^b^	68.85 ± 1.45^b^
	T2 (180)	74.23 ± 7.18^a^	67.59 ± 0.42^b^	66.93 ± 1.25^b^	63.63 ± 6.01^b^	64.06 ± 5.41^b^

Mean values in the same row with different lowercase were significantly different (*p* < 0.05). Mean values in the same column with different uppercase were significantly different (*p* < 0.05).

**Table 3:** Sensory acceptability of plain bread with different baking temperature and yam flour addition.

Color	Baking temperature (°C)	Bread U0 (0%)	Bread U1 (10%)	Bread U2 (15%)	Bread U3 (20%)	Bread U4 (30%)
Color	T1 (170)	3.48 ± 1.42^c^	3.02 ± 0.85^bc^	3.04 ± 0.89^bc^	2.88 ± 1.13^abc^	3.16 ± 1.40^bc^
	T2 (180)	3.40 ± 1.41^bc^	2.81 ± 1.04^ab^	2.84 ± 0.99^ab^	3.00 ± 1.04^bc^	3.28 ± 1.31^bc^
Aroma	T1 (170)	3.36 ± 1.15^a^	2.88 ± 1.05^a^	2.96 ± 1.06^a^	2.96 ± 1.14^a^	3.08 ± 0.91^a^
	T2 (180)	3.08 ± 1.15^a^	3.39 ± 1.03^a^	3.32 ± 0.95^a^	3.21 ± 0.97^a^	3.12 ± 1.24^a^
Texture	T1 (170)	3.76 ± 1.05^c^	3.28 ± 1.09^ab^	3.24 ± 1.05^ab^	3.20 ± 1.04^ab^	3.04 ± 1.21^a^
	T2 (180)	3.60 ± 0.96^bc^	3.39 ± 1.11^abc^	3.16 ± 1.11^abc^	3.25 ± 1.28^ab^	3.08 ± 1.04^ab^
Taste	T1 (170)	3.48 ± 1.16^ab^	2.96 ± 1.14^a^	3.56 ± 1.08^b^	3.40 ± 1.08^ab^	3.24 ± 1.09^ab^
	T2 (180)	3.32 ± 0.90^ab^	3.50 ± 1.19^ab^	3.04 ± 1.08^ab^	3.46 ± 0.96^ab^	2.96 ± 0.93^a^
Overall	T1 (170)	3.08 ± 1.23^ab^	3.60 ± 0.93^b^	2.80 ± 0.95^a^	3.32 ± 0.94^ab^	3.24 ± 1.09^ab^
	T2 (180)	3.16 ± 0.95^ab^	3.39 ± 1.12^ab^	3.40 ± 0.88^b^	3.13 ± 1.28^ab^	3.32 ± 1.10^ab^

Mean values in the same row with different lowercase were significantly different (*p* < 0.05). Mean values in the same column with different uppercase were significantly different (*p* < 0.05). 1: very disliked; 2: disliked; 3: liked; 4: preferred; 5: very liked. The letter above the bar graph beside the same number shows that it is not different based on the DNMRT statistical test at the level of confidence 5%.

## Data Availability

The data used and/or analyzed in the study are available from the corresponding author on reasonable request.
